# Coherent Structure in Indium Doped Phase Change Materials

**DOI:** 10.3390/ma18050934

**Published:** 2025-02-21

**Authors:** Rui Wang, Yonghui Zheng, Qianchen Liu, Tao Wei, Tianjiao Xin, Cheng Liu, Qiongyan Tang, Guangjie Shi, Bo Liu, Yan Cheng

**Affiliations:** 1 Key Laboratory of Polar Materials and Devices (MOE), Department of Electronics, East China Normal University, Shanghai 200241, China; 51254700107@stu.ecnu.edu.cn (R.W.); 52214700045@stu.ecnu.edu.cn (T.X.); 52274700031@stu.ecnu.edu.cn (C.L.); qytang@phy.ecnu.edu.cn (Q.T.); 51264700113@stu.ecnu.edu.cn (G.S.); 2 Chongqing Key Laboratory of Precision Optics, Chongqing Institute of East China Normal University, Chongqing 401120, China; 3 Suzhou Key Laboratory for Nanophotonic and Nanoelectronic Materials and Its Devices, School of Materials Science and Engineering, Suzhou University of Science and Technology, Suzhou 215009, China; liuqianchen2022@126.com (Q.L.); weitao@usts.edu.cn (T.W.); liubo@mail.usts.edu.cn (B.L.)

**Keywords:** phase change memory, indium doped Ge_2_Sb_2_Te_5_, thermal stability, coherent structure

## Abstract

Phase change memory (PCM) technology demonstrates significant potential as a next-generation non-volatile storage solution for information applications. Ge_2_Sb_2_Te_5_ (GST) alloy, the most well-established material employed in commercial PCM devices, exhibits limited thermal stability. Doping, as an effective approach for enhancing thermal stability, often induces element segregation and phase separation. This study systematically investigates the impact of indium (In) doping on GST phase-change material. Experimental results demonstrate that In doping significantly enhances the thermal stability of GST film. In17GST exhibits a 130 °C increase in crystallization temperature (from 181 °C to 311 °C). Especially, the introduction of In leads to the formation of In_2_Te_3_ phase, which exhibits a remarkably similar crystal structure to GST with only a ~2% lattice mismatch. Consequently, In_2_Te_3_ phase forms a coherent structure with GST lattice, thereby promoting the stability of the phase boundary. Additionally, In_2_Te_3_ phase facilitates efficient heating with a 5.7% improvement in heating efficiency (913 K vs. 864 K at 5 ns) and contributes to improved RESET operations in PCM devices. Our study lays the foundation for the composition and structure design for high thermal stability and low power consumption in PCM devices.

## 1. Introduction

With the development of modern information storage technology, the existence of “memory wall” and “power wall” has posed a serious challenge to the traditional von Neumann architecture [1]. Phase-change memory (PCM), a new storage technology that has achieved initial mass production, offers advantages such as fast read and write speeds, low operating power consumption, and good compatibility with CMOS technology [2,3]. Phase change memory (PCM) devices utilize phase change materials (PCMs) as their core storage medium. Acting as the crucial role in PCM, the microstructure of chalcogenide phase change materials strongly affects the practical storage performance. For instance, Ge_2_Sb_2_Te_5_ (GST) alloy is the most widely used phase change material owing to its fast-switching speed and good cycle endurance, while vacancy ordering process will drive a transition from the metastable face-centered cubic (fcc) phase to the hexagonal (hex) phase at a relatively moderate temperature, leading to a relatively low thermal stability [1,4,5,6]. Suppressing this transition is essential to maintain the metastable fcc phase and ensure reliable PCM operation. At present, researchers have never stopped trying to optimize the storage performance of GST-based phase change materials.

Beyond thermal stability, optimizing thermal conduction parameters to reduce melting time is also useful for PCMs. Strategies such as metal foams and nanoparticles [7,8,9,10] have been explored, but challenges like particle aggregation and cost limitations still remains. Doping is still an important means of improving both the resistance rate, data retention, cycle life, and crystallization speed of PCMs without changing the device or material structure [11]. To date, many elements have been selected as doping components to improve the storage performance of GST-based PCM devices [12,13,14,15,16,17]. Among them, In-doped GST (InGST) phase change materials have shown excellent thermal stability and lower power consumption [18,19,20]. Wuttig et al. claimed a small amount of crystalline In_2_Te_3_ in the crystallized In_0.3_Ge_2_Sb_2_Te_5_ alloy through X-ray diffraction (XRD) technology [18]. However, Song et al. found that In dopant occupies the cationic positions to form In-Te octahedrons through TEM technology [20]. Thus, the distribution of In dopant in GST alloy is still not fully understood, which obstructs the clarification of the regulation mechanism. Therefore, this work mainly focuses on the structural transition characteristics in InGST thin films to reveal the relationship between structural changes and electrical storage performance.

It was found that In doping can significantly improve the crystallization temperature of GST thin film, postpones the transition from face-centered cubic (fcc-) to hex-phase, and also effectively refine the crystallized nanograins. Furthermore, we found a zinc blende (zc-)-phase In_2_Te_3_ structure in the crystalline thin films, which exhibits good lattice matching with the matrix fcc-phase GST and forms a natural epitaxial superlattice structure along the (111) plane, which is beneficial to improve heating efficiency. Hence, the InGST material system is a potential candidate for high thermal stability and low power consumption applications after appropriate composition and structure design. This work demonstrates the coherent structure between In_2_Te_3_ and GST, enabled by lattice matching, which enhances thermal stability—a novel insight not previously reported.

## 2. Materials and Methods

InGST films with different indium doping concentrations (~200 nm thick) on a SiO_2_ substrate were deposited by magnetron co-sputtering using In and GST targets at room temperature, and the films for TEM observation were deposited on TEM grids with a thickness of 30 nm. The base pressure of the vacuum system is about 2 × 10^−4^ Pa. With the aid of energy dispersive spectroscopy (EDS) accessory (EDAX, Oxford, UK), the average atomic ratios of In element in InGST films were determined to be In_9_(Ge_2_Sb_2_Te_5_)_91_ and In_17_(Ge_2_Sb_2_Te_5_)_83_ (denoted as In9GST, In17GST), respectively. The sheet resistances of the deposited films as a function of temperature were in situ measured by a test equipment consists of a probe stage, a temperature controller and a resistance tester. And the thermal stability is evaluated with a ramp rate of 40 °C/min and a cooling rate of 100 °C/min n in vacuum chamber. The TEM bright-field (BF) images and corresponding selected area electron diffraction (SAED) patterns were obtained using the TEM mode of JEOL 2100F (JEOL Ltd. from Tokyo, Japan) under 200 kV. The in situ heating crystallization of InGST film was carried out in a heating holder (Gatan 628) in TEM. The STEM-BF images were captured using the STEM mode of JEM Grand ARM300F microscope (JEOL Ltd. from Tokyo, Japan) with double spherical aberration (Cs) correctors under 300 kV. The microstructure of the film was studied using XRD in an X-ray diffractometer (PANalytical X’Pert PRO, PANalytical B.V., Almelo, The Netherlands) with Cu Kα radiation source. The sheet resistances of the deposited films as a function of temperature were in situ measured by a test equipment consists of a probe stage, a temperature controller and a resistance tester. And the thermal stability is evaluated with a ramp rate of 40 °C/min and a cooling rate of 100 °C/min n in vacuum chamber. The TEM bright-field (BF) images and corresponding selected area electron diffraction (SAED) patterns were obtained using the TEM mode of JEOL 2100F under 200 kV. The in situ heating crystallization of InGST film was carried out in a heating holder (Gatan 628) in TEM. The STEM-BF images were captured using the STEM mode of JEM Grand ARM300F microscope with double spherical aberration (Cs) correctors under 300 kV. The microstructure of the film was studied using XRD in an X-ray diffractometer (PANalytical X’Pert PRO, PANalytical B.V., Almelo, The Netherlands) with Cu Kα radiation source. A T-shaped phase-change storage cell was built using multi-physical simulation software COMSOL v.5.6 to compare the heating efficiency of InGST and GST film under the same current stimulation.

## 3. Results

**Figure 1:**
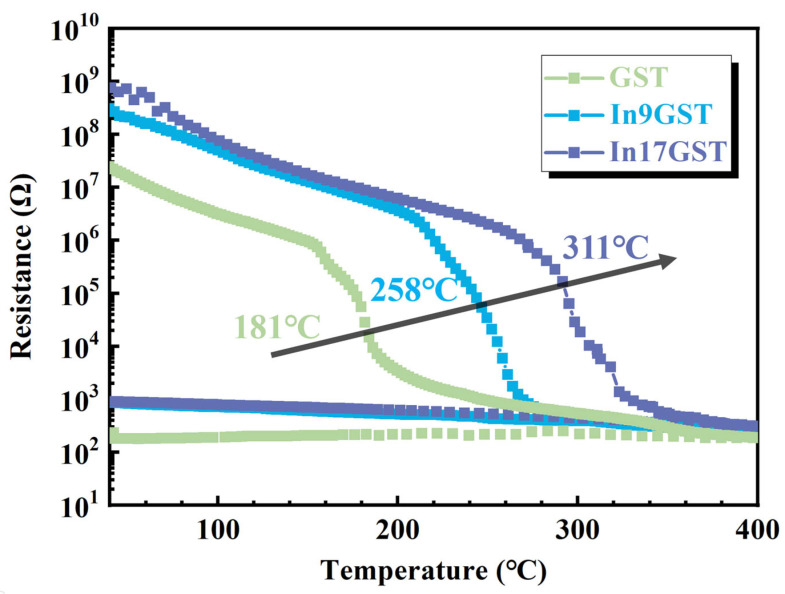
The resistance-temperature curves in GST, In9GST and In17GST films with a heating rate of 40 °C/min. The arrow indicates the increasing trend of crystallization temperature after In doping.

Figure 1 presents the resistance-temperature (R-T) curves of GST and InGST films at different temperatures (40–400 °C). As the temperature rises, the resistance decreases in GST film, showing a semiconductor-like behavior. Prior to 181 °C, the resistance gradually decreases from 4 × 10^7^ Ω to 1 × 10^6^ Ω. Around 181 °C, a sharp resistance decreasing from 10^6^ Ω to 10^4^ Ω can be observed, corresponding to the amorphous-to-fcc-phase transition in GST film. After In doping, the resistance at room temperature increases to ~1.7 × 10^9^ Ω. During the heating process, the crystallized temperature has postponed to ~258 °C for In9GST and even ~311 °C for In17GST as depicted by the black arrow. The increase in crystallization temperature indicates a better amorphous thermal stability after In doping. Additionally, both the crystalline and amorphous resistance in InGST film is higher than those of the GST, which is beneficial to generate more Joule heat to induce amorphization process in the practical devices at the same current.

**Figure 2:**
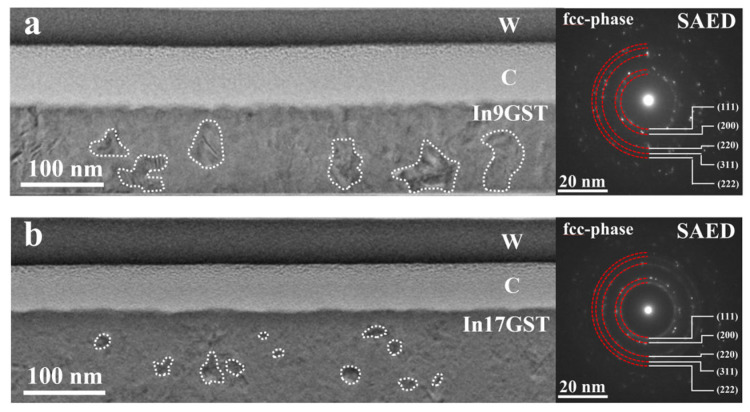
The crystalline microstructure of In9GST and In17GST film annealed at 300 °C. BF image and corresponding SAED patterns in (**a**) In9GST and (**b**) In17GST. From top to bottom, they are W (tungsten), C (carbon) and phase-change material, respectively. The dashed white lines denoted the identifiable nanograins.

To understand the microstructural transformation behavior of InGST film at different concentrations, the microstructure of the crystallized films annealed at 300 °C inside TEM were investigated as shown in Figure 2. For the In9GST film, the BF image demonstrated that it has crystallized into randomly distributed nanograins, and the size of some nanograins can reach ~50 nm (denoted by dashed white lines) in Figure 2a. Corresponding SAED pattern (right side of Figure 2a) shows discontinued diffraction rings and fits well with the fcc-phase. Nevertheless, compared with the in situ heating crystallization process in pure GST film, the crystallization process of the In9GST film after In doping has significantly slowed down, in which large hex-phase grains can be observed in the former when heated to 300 °C [21]. As for In17GST film, the grain size has further decreased at the same annealing temperature, and the size of some nanograins is only about 20 nm (denoted by dashed white lines) as shown in Figure 2b, and showing continued diffraction rings in the corresponding SAED pattern belongs to fcc-phase. Therefore, In element is an effective modifying element for improving the thermal stability of the GST film, and the higher the doping concentration, the better thermal stability and smaller grain size is obtained, which is beneficial to improve the reliability of the device.

**Figure 3:**
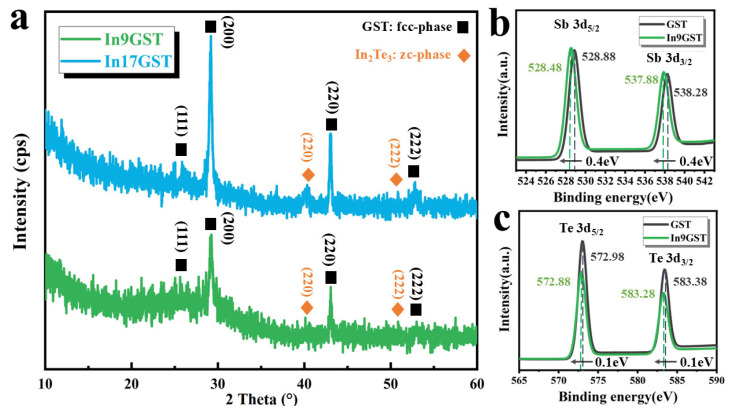
(**a**) X-ray diffraction (XRD) patterns of In9GST and In17GST film annealed at 400 °C, (**b**,**c**) X-ray photoelectron spectroscopy (XPS) patterns of GST and In9GST films annealed at 300 °C. The black arrows indicate the shift trend of the XPS peaks for Sb and Te element before (black dashed vertical line) and after (green dashed vertical line) doping.

Furthermore, X-ray diffraction (XRD) technology was used to study the structural transformation characteristics of In9GST and In17GST films from a more macroscopic perspective, Figure 3a depicts the XRD patterns of In9GST and In17GST films annealed at 400 °C. Clearly, the main peaks still belong the metastable fcc-phase with lattice parameter of a = 6.01 Å, in which (111), (200) and (220) peaks can be observed. Besides, additional crystalline peaks belong to zinc blende (zc-)-phase In_2_Te_3_ structure with lattice parameter of a = 6.16 Å were detected [22], and the higher the doping concentration, the more obvious these peaks are. To understand the phase separation phenomenon in InGST film, X-ray photoelectron spectroscopy (XPS) experiment was conducted to investigate the binding state variation in crystalline In9GST film, as shown in Figure 3b,c. In Figure 3b, it was found that the Sb-3d core level spectra shift towards lower energy (~0.4 eV) after In doping, demonstrating the variation of Sb chemical environment. Similar phenomenon also happens in Te element (~0.1 eV) as shown in Figure 3c. It is known that electro-negativity of In (1.78) is smaller than that of Sb (2.05) and Te (2.12), hence the decrease of binding energy suggests that the In element (163 pm) would serve as cationic impurities to replace some Ge (122 pm) and Sb (145 pm) atoms in the crystal lattice, which strongly affects the chemical environment of the constituent elements. One may note the chemical environment of the Sb element changes more dramatically than Te element, which suggests that the elements with lower content are more sensitive to doping elements. Similar XPS was previously reported by Wang et al., and they direct observe a well geometrically match between In-Te, host Ge-Te and Sb-Te octahedrons through atomic EDS mapping [20]. Besides, a larger electro-negativity difference (∆S) of In-Te than Sb-Te is also beneficial to generate more grain boundaries to inhibit the grain growth as observed in Figure 2 [23].

**Figure 4:**
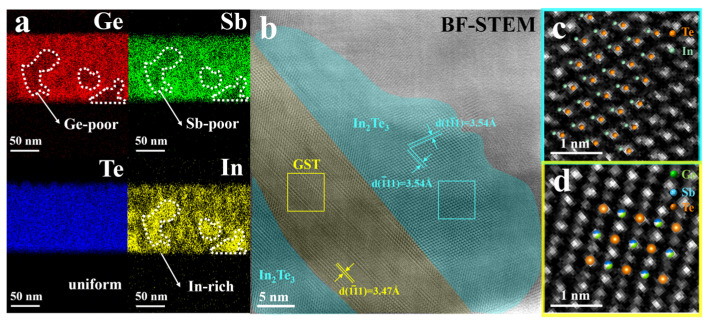
(**a**) EDS mappings of elements Ge, Sb, Te and In, (**b**) Bright field resolution transmission electron microscopy (BF-TEM) of In9GST, (**c**) HAADF images of In_2_Te_3_ and (**d**) GST.

We have observed the appearance of zc-phase In_2_Te_3_ peak in XRD spectrum. While previous results claimed that In element has entered the crystal lattice. Then one question naturally arise, does the zc-phase In_2_Te_3_ really exists in the crystalline film. To clarify this question, we further investigated the element distribution and atomic arrangement in the crystalline microstructure in InGST film. Figure 4a shows the EDS mappings of elements Ge, Sb, Te and In annealed at 400 °C in In9GST film. Clearly, the distribution of Te element is uniform, while Ge, Sb and In element is nonuniform. More specially, in areas where the Ge and Sb element signals are weaker, the In element signal is stronger, and vice versa. The STEM-BF image [24] in Figure 4b is the enlargement of the In-enriched region. Inspecting the atomic arrangement of a large grain in the bottom left area of Figure 4b, we can find that the middle area belongs to the fcc-phase GST, and the left and right areas belong to zc-phase In_2_Te_3_, whose atomic arrangement is depicted in Figure 4c,d. Hence, this result is consistent with the XRD spectrum in Figure 3. Although nanoscale phase separation occurs, the lattice parameters of the zc-phase In_2_Te_3_ and the fcc-phase GST both still belong to cubic crystal structure, and the lattice parameter difference between the two structures are less than 3%. Figure 4b also demonstrates that the (111) lattice plane of zc-phase In_2_Te_3_ grains is parallel to (111) lattice of fcc-phase GST, that is to say a natural superlattice-like structure has formed along the (111) plane of fcc-phase GST. Previous literature has reported that although the lattice parameters of TiTe_2_ and Sb_2_Te_3_ deviate by 11%, the phase change heterostructure based on TiTe_2_ and Sb_2_Te_3_ shows good lattice matching, ultra-low power consumption, and ultralow noise and drift for memory operation [25]. Therefore, even though nanophase separation occurs in InGST, this superlattice-like structure shows good promising to achieve low power application.

**Figure 5:**
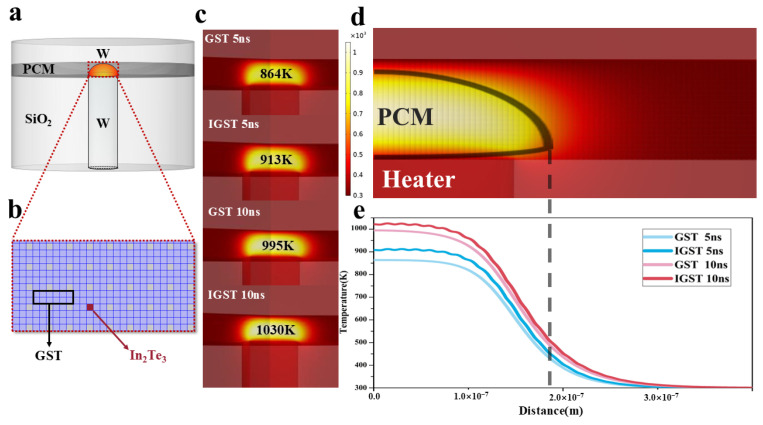
(**a**) T-type phase-change storage unit simulated by COMSOL. (**b**) Enlarged view of the structure of phase change material layer. (**c**) Temperature distribution of T-type phase-change memory device under the same current excitation of 5 ns and 10 ns. (**d**) Maximum temperature in the layer of GST and InGST phase change material when the same amplitude current pulse is applied to excite 5 ns and 10 ns. (**e**) The simulated temperature-radial distance curve in GST and InGST device at the same central vertical height with different timescale. The region to the left of the gray line experiences a significant temperature rise during the electrical operation process.

To further study the impact of nanoscale phase separation on the practical device performance, we used the finite element method (FEM) to simulate and compare the difference in T-shape storage device heating efficiency before and after doping, as shown in Figure 5. Figure 5a is the overall schematic structure of the T-shape device. The thickness of phase change material layer is 100 nm, which are divided into grid shape to represent the poly-crystalline morphology. Each grid (5 nm × 5 nm) denotes the small crystal grain as shown in Figure 5b. For doped InGST film, ~11% of grids are orderly chosen to be the zc-In_2_Te_3_ grains, and the other parts of grids are GST grains. Note that some In atoms have enter the crystal lattice of GST grains, since we mainly consider the effect of nanoscale phase separation on the T-shape device, we use the physical parameters of In_2_Te_3_ and GST to conduct the FEM simulation. The Joule heat is mainly generated in the phase change films. The thermal transfer obeys the standard heat conduction equation [26]:

(1) ∇⋅κ∇T+Q=ρc∂T/∂t

where κ is the thermal conductivity, c is the specific heat, ρ is the density, t is the time, T is the temperature, and Q is the Joule heat per unit volume and per unit time, which is called the heat density. The relevant physical parameters involved include the material’s density, specific heat capacity, thermal conductivity, and electrical conductivity. Among them, the density of GST is 6930 Kg/m^3^ [27], the specific heat capacity is 202 J/Kg·K [28], the thermal conductivity is 0.45 W/m·K [29], and the electrical conductivity is 1 × 10^5^ Ω^−1^·m^−1^ [30]. The density of In_2_Te_3_ is 5850 Kg/m^3^ [31], the specific heat capacity is 201 J/Kg·K [27], the thermal conductivity is 1.255 W/m·K [32], and the electrical conductivity is 4 × 10^3^ Ω^−1^·m^−1^ [31]. Constant current pulse was applied to the T-shaped storage cell. Natural convection and radiation effects were excluded in simulations due to the microscale device dimensions and short pulse duration (5–10 ns). However, in large-scale applications, these factors may influence heat dissipation and require consideration. It can be observed that the highest temperature after 5 ns for GST and InGST is 864 K and 913 K, indicating that the heating efficiency in InGST is higher than that of GST due to the superlattice-like structure [31]. At 10 ns, the heating efficiency in InGST is still better than that of GST. In_2_Te_3_’s higher thermal conductivity improves heat localization. Moreover, In_2_Te_3_ has a lower conductivity, according to Equation (1), a lower conductivity generates more heat at the same current density, thus leading to improved RESET performance and more efficient heating in PCM devices. We also compared the temperature distribution profile in the central area, as shown in Figure 5d,e. The temperature of the PCM decreases gradually with the increase of the radial distance, and the temperature of the InGST layer at the same radial distance is always higher than that of the GST layer, therefore lower energy is needed for the storage operation in InGST film due to the ordered superlattice-like nanostructure.

## 4. Discussion

The integration of indium (In) doping into Ge_2_Sb_2_Te_5_ (GST) phase-change materials offers significant improvements in thermal stability and power efficiency, primarily through the formation of coherent In_2_Te_3_/GST interfaces. Our R-T resistance curve and subsequent microstructure investigation unambiguously demonstrated that In doping postponed crystallization to 311 °C (vs. 181 °C for GST), significantly improving the crystallization temperature of GST thin film, suppressing the transition from fcc-to-hex phase during the heating process. Furtherly, via XRD (Figure 3a), TEM (Figure 4b), and EDS mapping (Figure 4a), a zc-phase In_2_Te_3_ were observed in the crystalline film, which enhance the thermal stability of GST films attributed to refined grains and In_2_Te_3_ phase stabilization. The ~2% lattice mismatch between In_2_Te_3_ (a = 6.16 Å) and GST (a = 6.01 Å) enables coherent interfaces along (111) planes, forming a natural superlattice-like structure with fcc-phase GST, which is a favored nanostructure in phase change material system to achieve low power consumption and low resistance drift application. Hence, this work provides important inspiration for the composition and structure design for high thermal stability and low power consumption in PCM devices.

## 5. Conclusions

In conclusion, this study demonstrates that indium doping significantly enhances the performance of GST-based phase-change memory devices by forming coherent In_2_Te_3_/GST interfaces, which improve thermal stability, power efficiency, and structural integrity. The ~2% lattice mismatch between In_2_Te_3_ and GST enables low-defect epitaxial growth, delaying hexagonal phase formation and reducing RESET energy consumption. However, challenges such as dopant segregation, process scalability, and material costs must be addressed to enable industrial adoption. Future research should explore co-doping strategies, advanced fabrication techniques, and alternative dopants to optimize performance while reducing costs. By addressing these challenges, In-doped GST systems hold great promise for advancing next-generation, low-power phase-change memory technologies.

## Data Availability

The original contributions presented in this study are included in the article. Further inquiries can be directed to the corresponding authors.
